# A phase 2 randomized dose-ranging study of the JAK2-selective inhibitor fedratinib (SAR302503) in patients with myelofibrosis

**DOI:** 10.1038/bcj.2015.63

**Published:** 2015-08-07

**Authors:** A Pardanani, A Tefferi, C Jamieson, N Y Gabrail, C Lebedinsky, G Gao, F Liu, C Xu, H Cao, M Talpaz

**Affiliations:** 1 Division of Hematology, Department of Medicine, Mayo Clinic, Rochester, MN, USA; 2 Department of Medicine, UCSD Moores Cancer Centre, University of California San Diego, La Jolla, CA, USA; 3 Gabrail Cancer Center, Canton, OH, USA; 4 Sanofi Oncology, Sanofi, Cambridge, MA, USA; 5 Division of Hematology-Oncology, Department of Internal Medicine, Comprehensive Cancer Center, The University of Michigan Hospital and Health Systems, Ann Arbor, MI, USA

## Abstract

In this phase 2 open-label randomized study, 31 patients with intermediate-2 or high-risk myelofibrosis received fedratinib 300, 400 or 500 mg once daily in consecutive 4-week cycles. Mean spleen volume reductions at 12 weeks (primary end point) were 30.3% (300 mg), 33.1% (400 mg) and 43.3% (500 mg). Spleen response rates (patients achieving ⩾35% spleen reduction) at 12/24 weeks were 30%/30% (300 mg), 50%/60% (400 mg) and 64%/55% (500 mg), respectively. By 4 weeks, improvements in myelofibrosis (MF)-associated symptoms were observed. At 48 weeks, 68% of patients remained on fedratinib and 16% had discontinued because of adverse events (AEs). Common grade 3/4 AEs were anemia (58%), fatigue (13%), diarrhea (13%), vomiting (10%) and nausea (6%). Serious AEs included one case of reversible hepatic failure and one case of Wernicke's encephalopathy (after analysis cutoff). Fedratinib treatment led to reduced STAT3 phosphorylation but no meaningful change in *JAK2*V617F allele burden. Significant modulation (*P<*0.05, adjusted for multiple comparisons) of 28 cytokines was observed, many of which correlated with spleen reduction. These data confirm the clinical activity of fedratinib in MF. After the analysis cutoff date, additional reports of Wernicke's encephalopathy in other fedratinib trials led to discontinuation of the sponsored clinical development program.

## Introduction

Myelofibrosis (MF) is a *BCR–ABL1*-negative, chronic myeloproliferative neoplasm (MPN) that may develop *de novo* (that is, primary MF (PMF)) or from fibrotic transformation of pre-existing polycythemia vera (PV) or essential thrombocythemia (ET).^1^ MF involves the transformation and clonal proliferation of hematopoietic stem/progenitor cells and dysregulation of their associated cytokine signaling pathways.^2^ Patients typically present with cytopenias, splenomegaly and burdensome constitutional symptoms.^3^ Although life expectancy is normal in patients with World Health Organization (WHO)-defined ET, it is decreased in those with PV and severely compromised in PMF.^4^ Median survival times in patients with intermediate-2 or high-risk PMF are 48 and 27 months, respectively.^5^

Constitutive activation of the Janus kinase (JAK)-signal transducer and activator of transcription (STAT) signaling pathway is a hallmark feature of MF and is often associated with somatic mutations of the *JAK2* and *MPL* genes.^6^ Almost all patients with PV, and 50–60% of patients with ET and PMF, carry the *JAK2*V617F mutation.^7, 8, 9^ The *MPL*W515 mutation is uncommon in PV^10^ but present in 5–10% of patients with ET and PMF.^11, 12^ Animal model data suggest that constitutive activation of JAK-STAT signaling can drive abnormal hematopoiesis and may be sufficient for full development of the MPN phenotype.^13^ Aberrant JAK-STAT signaling may also lead to dysregulation of inflammatory cytokines/chemokines, thought to underlie many of the debilitating symptoms associated with MF.^14^ The JAK1/JAK2 inhibitor ruxolitinib is approved for the treatment of intermediate and high-risk MF, based on improvement of splenomegaly and disease-related symptoms.^15, 16^

Fedratinib (SAR302503/TG101348) is a potent JAK2-selective inhibitor with activity against cells expressing *JAK2*V617F and *MPL*W515L in MPN disease models.^17, 18, 19^ In clinical trials, fedratinib treatment of patients with intermediate-2 or high-risk MF effectively reduced spleen size and disease-related symptoms, regardless of *JAK2* mutational status.^20, 21^ In a phase 1 dose escalation study, the lowest fedratinib once-daily dose associated with clinical activity was 240 mg and the maximum tolerated dose was 680 mg.^20^ At doses above 520 mg, there was a trend toward increasing transfusion dependence over the 24-week study period. Based on these results, the current phase 2 study (ARD11936) was conducted to further explore the clinical activity, safety, pharmacokinetics (PKs) and pharmacodynamics (PD) of fedratinib administered once daily at three doses (300, 400 and 500 mg) in patients with MF.

## Materials and methods

### Patients

Eligible patients were at least 18 years of age with a diagnosis of PMF, post-PV MF or post-ET MF, according to the 2008 WHO criteria.^22^ Other inclusion criteria included intermediate-risk level 2 or high-risk MF (International Working Group-Myeloproliferative Neoplasms Research and Treatment criteria),^5^ Eastern Collaborative Oncology Group performance status ⩽2, splenomegaly (palpable ⩾5 cm below the costal margin) and platelet counts ⩾50 × 10^9^/l. Patients were enrolled regardless of *JAK2* mutational status. Key exclusion criteria included splenectomy and previous treatment with a JAK2 inhibitor or any chemotherapy at any time before study entry, and immunomodulatory therapy or immunosuppressive therapy 14 days before treatment.

### Study design

This was a phase 2, randomized, open-label study, conducted at four centers in the United States. Patients were randomized (1:1:1) to receive fedratinib at doses of 300, 400  or 500 mg once daily, in consecutive 4-week cycles. An interactive voice response system was used for randomization. Patients received up to six cycles (24 weeks) of treatment. Thereafter, patients who continued to derive clinical benefit could remain on treatment until disease progression or unacceptable toxicity. At 24 weeks, patients in the 300 mg group were eligible for dose escalation up to 500 mg/day (100 mg/day increments) if there was a lack of adequate efficacy response and no safety concerns. Dose escalation was not permitted for patients in the 400  and 500 mg groups.

Patients were evaluated every 2 weeks during the first three cycles of treatment, at the beginning and end of each subsequent cycle, and 30 days after treatment discontinuation. The criteria for stopping or adjusting treatment are listed in the Supplementary Information.

### Study end points

The primary efficacy end point was percentage change in spleen volume based on magnetic resonance imaging (MRI) at 12 weeks (end of cycle 3) relative to baseline. Secondary end points included percentage change in spleen volume at 24 weeks (end of cycle 6); proportion of patients who achieved a spleen response (⩾35% reduction in spleen volume from baseline) at 24 weeks; duration of spleen response; symptom response (in patients with symptoms present at baseline, a 2-point improvement or resolution of that symptom) at weeks 4, 12 and 24, and end of therapy, as measured by the Myeloproliferative Neoplasm Symptom Assessment Form (MPN-SAF)^23^; PK/PD; and safety. Exploratory end points included the proportion of patients with baseline leukocytosis or thrombocytosis who achieved normalization of leukocyte and platelet counts, respectively, and change in transfusion requirements from baseline. *Ad hoc* analyses included symptom response rate (proportion of patients with ⩾50% reduction in total symptom score (TSS: sum of scores of the six key symptoms (early satiety, abdominal pain, abdominal discomfort, bone pain, night sweats and pruritus) calculated at each visit)); spleen response at 48 weeks; and comparison of health-related quality of life to week 24.

### Efficacy assessments

Spleen volume was assessed using MRI at a central imaging laboratory. Reviewers were blinded to fedratinib dose. Duration of spleen response was defined as the time from the date of the first response (⩾35% reduction in spleen volume from baseline by MRI) to the date of disease progression or death. In the absence of disease progression or death before the analysis cutoff date (28 November 2012), duration of response was censored at the date of the last valid assessment.

Patient-reported outcomes were assessed using the MPN-SAF^23^ and EuroQol 5-Dimensions (EQ-5D)^24^ questionnaires. The MPN-SAF assessed the presence and severity of fatigue (brief fatigue inventory)^25^ and 17 additional MPN-associated symptoms over the previous week on a scale from 0 (absent) to 10 (worst imaginable). The scores for the six key symptoms were evaluated individually and summated to determine the TSS at each visit.

### PKs and PDs

Fedratinib concentrations in plasma samples were determined using validated liquid chromatography-tandem mass spectrometry. Methods for the assessments of PK, *JAK2*V617F allele burden and phospho-STAT3 (pSTAT3) levels are described in the Supplementary Information. The plasma levels of 97 cytokines were measured using microsphere-based immunomultiplex assays (Rules Based Medicine, Austin, TX, USA) at baseline and up to week 12. Sample collection and data analysis are detailed in the Supplementary Information.

### Safety

Adverse events (AEs) were recorded and coded according to National Cancer Institute Common Terminology Criteria for Adverse Events version 4.03 (http://evs.nci.nih.gov/ftp1/CTCAE/CTCAE_4.03_2010-06-14_QuickReference_5x7.pdf).

### Statistical analyses

Descriptive statistics were calculated. No formal determination of sample size was performed and no formal statistical comparisons were made between the dose groups. Analysis of the primary efficacy end point was performed using the intent-to-treat (ITT) population (all randomized patients). Symptom response was assessed in ITT patients with evaluable baseline symptoms. The PK and PD populations comprised patients for whom evaluable data were available and who had received at least one complete cycle of fedratinib. Safety was assessed in ITT patients who had received at least one dose of fedratinib.

### Study oversight

The study was conducted according to the principles of the International Conference on Harmonisation guidelines for Good Clinical Practice. Written informed consent was obtained from all study participants. The study was approved by the appropriate local institutional review boards.

## Results

### Patient disposition and baseline demographics

Between 2 September and 21 December 2011, 31 patients were randomized to receive fedratinib at doses of 300 mg (*n=*10), 400 mg (*n=*10) or 500 mg (*n=*11). All patients received at least one dose of treatment. At the analysis cutoff date, 21 patients (68%) remained on treatment. The reasons for treatment discontinuation were AEs (300 mg (*n=*2), 400 mg (*n=*2), 500 mg (*n=*1)); withdrawal of consent (300 mg (*n=*2)); investigator discretion (300 mg (*n=*1)); withholding of drug for>8 weeks (500 mg (*n=*1)); and patient's personal reasons (300 mg (*n=*1)).

Patient baseline demographics and disease characteristics are described in Table 1; the 500 mg group had a smaller median spleen volume relative to the other groups. The most prevalent prior treatment for MF was hydroxyurea (20/31 patients (65%)).

### Efficacy

#### Spleen response

**Figure 1:**
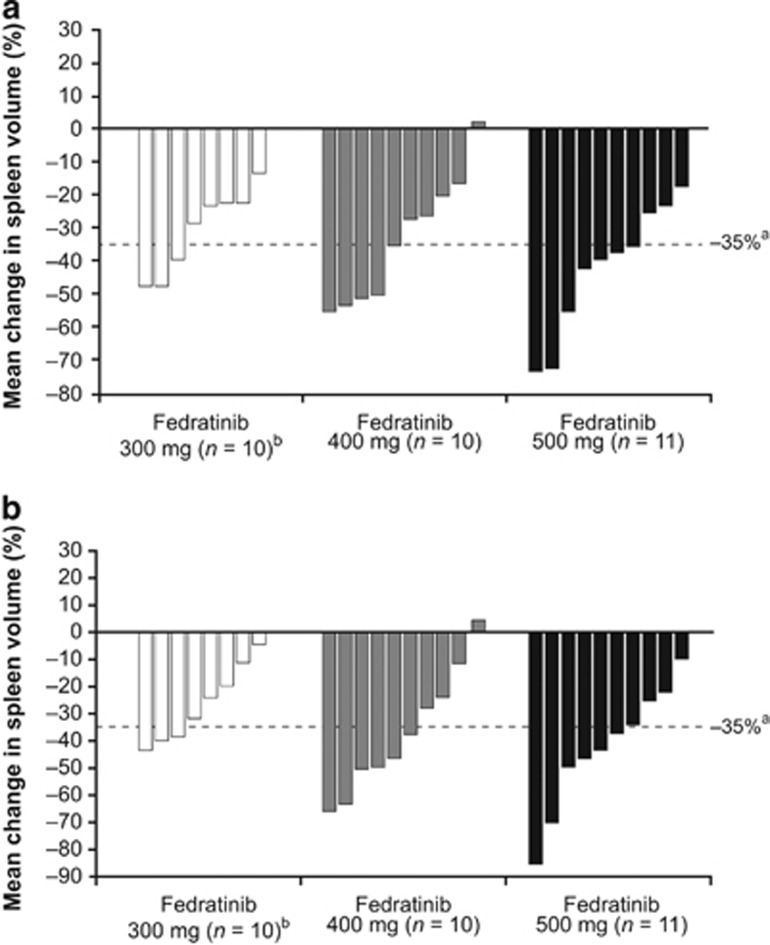
Waterfall plot of changes in spleen volume for individual patients at (**a**) 12 weeks and (**b**) 24 weeks. ^a^Patients had a spleen response if they had a ⩾35% reduction in spleen volume from baseline to week 24. ^b^Two patients discontinued treatment before week 12.

At week 12, the mean (s.d.) reduction in spleen volume relative to baseline (primary end point) for the ITT population was 30.3% (12.6%), 33.1% (19.0%) and 43.3% (19.0%) in the 300, 400 and 500 mg dose groups, respectively (Table 2). The reductions in spleen volume were similar at weeks 12 and 24 (Table 2). The waterfall plot (Figure 1) shows the changes in spleen volume for individual patients at weeks 12 and 24.

Spleen response (⩾35% reduction in spleen volume from baseline) rates over time are shown in Table 2. Responses in the 300 mg group were consistently lower than in the 400 and 500 mg groups. For two of the seven patients in the 500 mg group with a response at week 12, the reduction in spleen volume from baseline decreased below the 35% threshold between weeks 12 and 48; one of these patients had their fedratinib dose reduced to 200 mg/day. Among the patients who achieved a spleen response at any time point, the median (range) duration of response was 255 (253–270) days, 251 (1–256) days and 251 (82–253) days in the 300, 400 and 500 mg groups, respectively. These median values likely underestimate the total response duration because the data were censored at the analysis cutoff date.

#### Symptom response and other patient-reported outcomes

**Figure 2:**
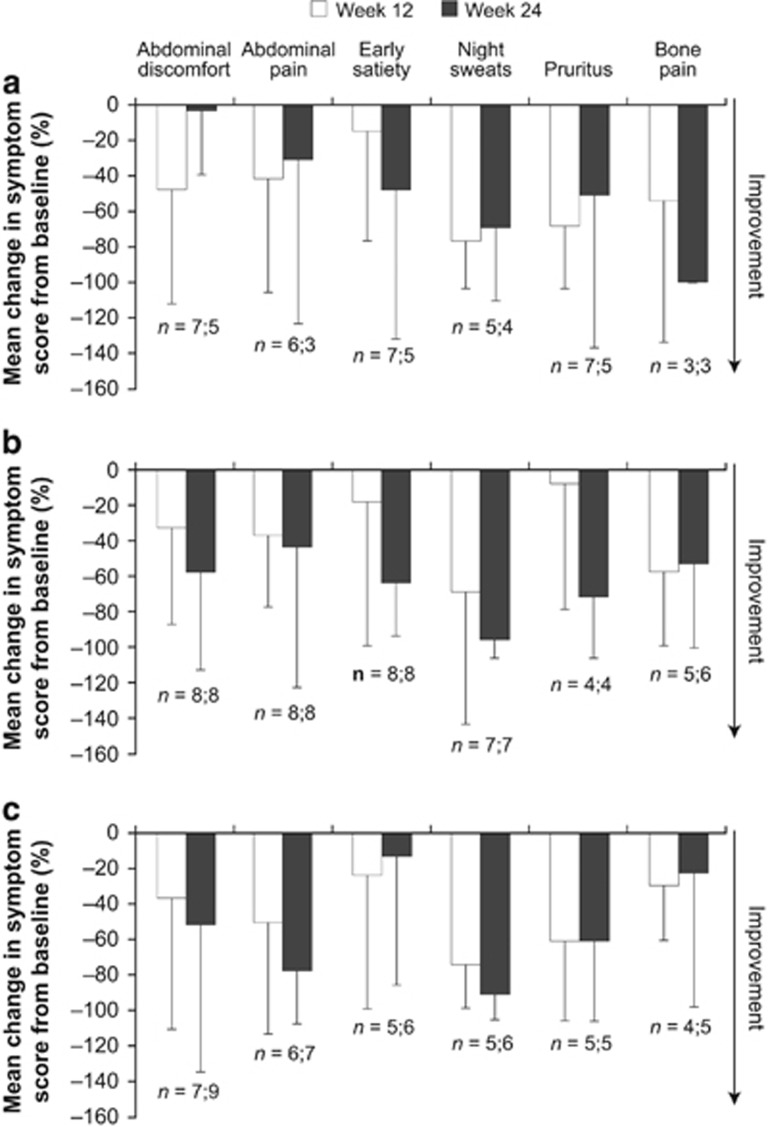
Symptom responses at weeks 12 and 24. Mean percentage change in score of the six key individual symptoms from baseline in the (**a**) 300 mg group, (**b**) 400 mg group and (**c**) 500 mg group. Numbers of evaluable patients (week 12; week 24) are denoted at the end of the bars. Error bars represent s.d. from the mean.

Mean scores for all six key MF symptoms were reduced from baseline at 12 weeks, with the greatest improvement seen for night sweats (Figure 2). Compared with week 12, reductions were broadly similar at week 24 for the six symptoms (Figure 2). The mean (s.d.) reduction in usual fatigue from baseline at 24 weeks was 18.0% (47.4%), 39.3% (38.1%) and 14.6% (56.9%) in the 300, 400 and 500 mg dose groups, respectively. Among ITT patients with evaluable baseline symptoms, TSS improved from baseline in all dose groups by week 4. The proportions of patients with a ⩾50% reduction in TSS at 4 weeks were 44% (4/9 patients), 50% (5/10) and 50% (4/8) in the 300, 400 and 500 mg groups, respectively. Corresponding rates at 24 weeks were 33% (3/9 patients), 60% (6/10) and 38% (3/8).

Among evaluable ITT patients, median EQ-5D score improved from baseline to 24 weeks for both the 400 mg (+5.0 (range: –55.0 to 30.0); *n=*7) and 500 mg (+5.00 (–50.0 to 50.0); *n=*7) groups. The EQ-5D median score did not improve in the 300 mg group over this period (–1.0 (–15.0 to 30.0); *n=*5).

#### Blood count changes

Among patients with baseline leukocytosis, 29% (2/7 patients), 44% (4/9) and 44% (4/9) in the 300, 400 and 500 mg groups, respectively, achieved normalization of leukocyte counts at 24 weeks. Five of 12 patients with thrombocytosis at baseline achieved platelet count normalization at 24 weeks (1/3, 4/4 and 0/5 patients in the 300, 400 and 500 mg groups, respectively). In the 300 and 400 mg groups, mean hemoglobin levels reached nadir following approximately 12–16 weeks of treatment, and then tended to increase up to week 48 (Supplementary Figure S1). Mean hemoglobin levels were lower in the 500 mg group than the other dose groups during treatment. Both patients in the 500 mg group who were red blood cell transfusion dependent at baseline remained transfusion-dependent post-baseline. Six patients (19%) who were transfusion independent at baseline became transfusion dependent on study (300 mg (*n=*1); 400 mg (*n=*3); 500 mg (*n=*2)).

### Pharmacokinetics

**Figure 3:**
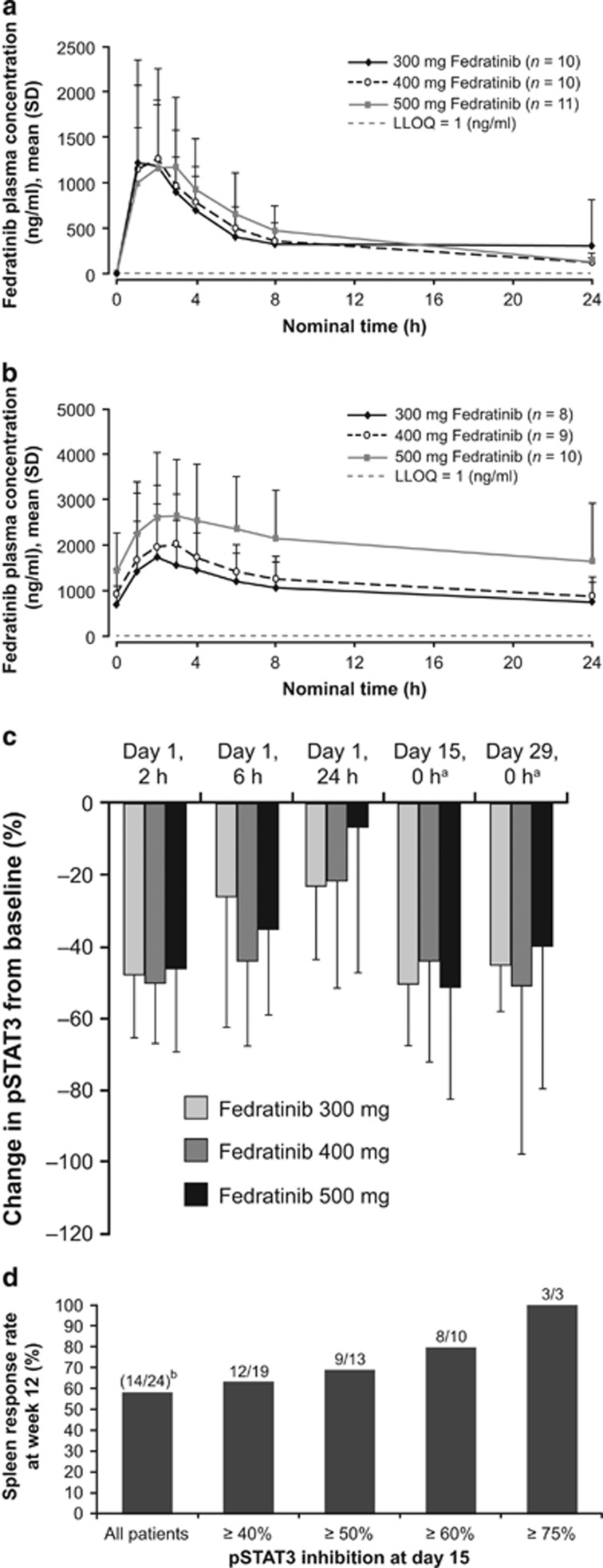
Fedratinib PKs and PDs. Fedratinib plasma concentration over 24 h at (**a**) day 1 and (**b**) day 29. (**c**) Percentage change in STAT3 phosphorylation from baseline during treatment (ITT population). (**d**) Correlation between pSTAT3 inhibition at day 15 and spleen response at week 12. ^a^A single sample was taken post-dose at days 15 and 29, although this had little impact on mean or median pSTAT3 levels. ^b^Number of spleen responders/numbers of pSTAT3 responders. LLOQ, lower limit of quantification; pSTAT3, phospho-STAT3; STAT3, signal transducer and activator of transcription-3.

The median peak plasma concentration (t_max_) of fedratinib on day 1 was reached approximately 2 h after the initial dose (Figure 3a). Fedratinib levels reached steady state by day 15. Fedratinib exposure (peak plasma concentration (C_max_) and area under the concentration–time curve from baseline to 24 h (AUC_0–24_]) largely overlapped across all three doses on day 1, and increased in a dose-related manner at steady state (day 29) (Figures 3a and b). At day 29, drug accumulation was 2.95- to 3.88-fold higher compared with day 1, based on the AUC_0–24_ comparisons.

### Pharmacodynamics

With all fedratinib doses, pSTAT3 levels were reduced relative to baseline. The greatest reductions on day 1 occurred 2 h post-dose, corresponding to the t_max_ of fedratinib. Inhibition of pSTAT3 then decreased at 6 and 24 h, concurrently with reduced fedratinib exposure (Figure 3c). The mean (s.d.) reductions in pSTAT3 from baseline at 2 h after the first dose of fedratinib were 47.9% (17.6%), 50.3% (16.9%) and 46.4% (22.5%) in the 300, 400 and 500 mg groups, respectively; similar levels of pSTAT3 inhibition were observed at fedratinib trough level at steady state (days 15 and 29). Patients with greater levels of pSTAT3 inhibition were more likely to achieve a spleen response (Figure 3d).

### *JAK2*V617F allele burden

Among 29 patients with an available baseline sample, 26 (90%) were *JAK2*V617F positive. Although the study was not powered to show a statistically significant reduction in *JAK2*V617F allele burden, there was no marked difference between baseline and week 48 across all three doses (Supplementary Table S1). At 48 weeks, the median (range) changes in allele burden were –4.57% (–24.1 to 0.5%), –0.05% (–21.9 to 2.0%) and –8.16% (–68.8 to 63.0%) in the 300, 400 and 500 mg groups, respectively. At 24 weeks, 14/25 patients (56%) with baseline *JAK2*V617F mutation achieved a spleen response. Of the three patients with wild-type *JAK2*, two patients had spleen volume reductions of <35% (one each with *MPL*W515K and *MPL* wild type).

### Regulation of cytokine expression

Plasma levels changed significantly from baseline (⩾1.5-fold change (*P<*0.05), adjusted for multiple comparisons) over the first 12 weeks of treatment for 28 of the 97 cytokines measured (Supplementary Table S2). Four cytokines were consistently upregulated and 15 downregulated at weeks 4, 8 and 12. Erythropoietin, ferritin, adiponectin and leptin had the highest level of upregulation; C-reactive protein, T-cell-specific protein RANTES (regulated upon activation normal T-cell expressed) and EN-RAGE (extracellular newly identified receptor for advanced glycation end products binding protein) were most strongly downregulated (Supplementary Table S2).

**Figure 4:**
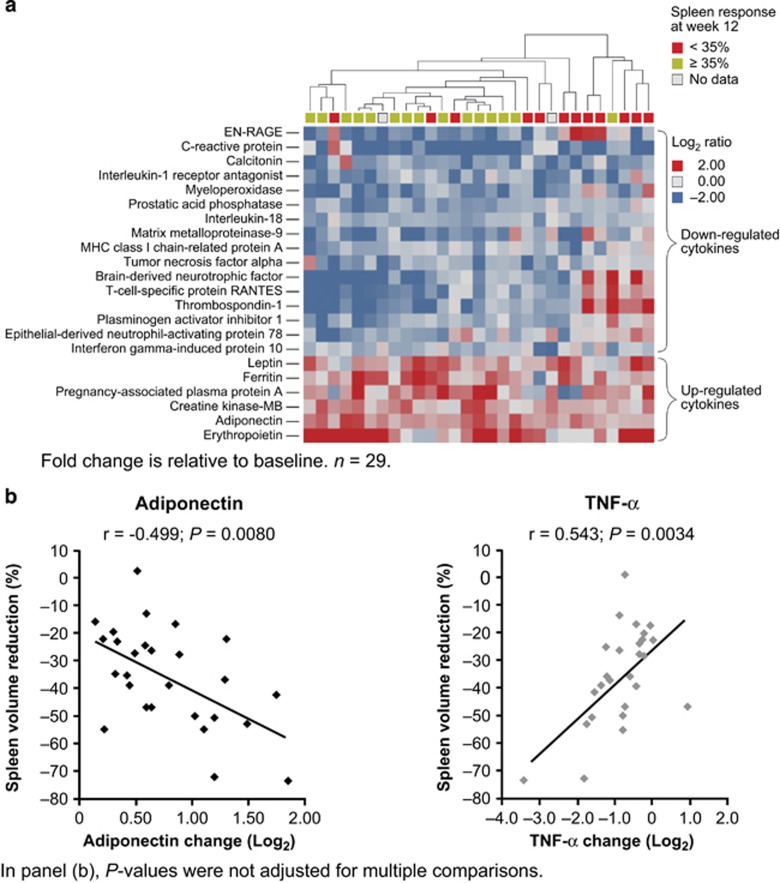
Cytokine regulation by fedratinib. (**a**) Hierarchical clustering of patients by changes in the 22 regulated cytokines at week 4. (**b**) Correlation between changes in levels of adiponectin and TNF-α and reduction in spleen volume. MHC, major histocompatibility complex.

Hierarchical clustering of patients according to changes in cytokine expression indicated that patients with a spleen response at 12 weeks had a similar pattern of cytokine regulation at 4 weeks (Figure 4a). Of the cytokines showing a ⩾1.5-fold change at 12 weeks, 13 were significantly correlated (*P<*0.05, not adjusted for multiple comparisons) with spleen volume reduction at this time point (Supplementary Table S3). Using the more stringent adjusted *P*-value (adjusted for false discovery rate), eight of these cytokines met the criteria for statistical significance (*P<*0.05): prostatic acid phosphatase, tumor necrosis factor-α (TNF-α), myeloperoxidase, interleukin-18, matrix metalloproteinase-9, adiponectin, carcinoembryonic antigen and creatinine kinase-MB. Representative data for adiponectin and TNF-α are plotted in Figure 4b. Changes in adiponectin correlated significantly with spleen volume reduction at week 4 (correlation coefficient (*r*)=–0.407; *P=*0.035); week 8 (*r*=–0.505; *P=*0.0073) and week 12 (*r*=–0.499; *P=*0.0080).

### Safety

Five patients discontinued treatment because of a treatment-emergent AE (TEAE) (300 mg: grade 3 anemia (*n=*1), grade 4 hepatic failure, which resolved with drug discontinuation (*n=*1); 400 mg: grade 3 dyspnea and grade 4 leukocytosis (*n=*1), intermittent grade 2 nausea (1); 500 mg: grade 3 anemia and grade 4 thrombocytopenia (*n=*1)).

The median numbers of cycles administered per patient were 12.0 (range: 2–16), 14.0 (6–16) and 13.0 (7–17) for the 300, 400 and 500 mg groups, respectively. No patient in the 300 mg group had dose escalation up to data cutoff. At least one dose reduction was required in 6/10, 8/10 and 10/11 patients in the 300, 400 and 500 mg dose groups, respectively, and most reductions were of one dose level; two patients in the 400 mg group had two dose reductions to 200 mg, and one patient in the 500 mg group had three dose reductions to 200 mg. The most common reasons for dose reduction were anemia (*n=*5) and elevated lipase levels (*n=*4).

All patients had at least one TEAE. Grade 3/4 TEAEs were reported in 8/10 patients in the 300 and 400 mg groups, and in all patients in the 500 mg group. The most common nonhematologic TEAEs were gastrointestinal disorders (diarrhea, nausea, vomiting and constipation), fatigue, peripheral edema, dyspnea and pain in extremity (Table 3). Most (81%) gastrointestinal toxicities were grade 1/2. Four patients required dose reductions because of gastrointestinal toxicities, and one of these patients subsequently discontinued treatment because of grade 2 nausea. The prevalence of gastrointestinal TEAEs decreased over time (Supplementary Figure S2). The most frequent hematologic abnormality was anemia, seen in all 31 patients (grade 3/4 58% (18/31); Table 3). The overall prevalence of thrombocytopenia was 55% three patients developed grade 3 thrombocytopenia (300 mg (*n=*2); 500 mg (*n=*1)) and two patients had grade 4 thrombocytopenia (*n=*1 in each of the 400 mg and 500 mg groups). Grade 1/2 neutropenia occurred in four patients (300 mg (*n=*2); 500 mg (*n=*2)); no grade 3/4 neutropenia was reported. In most cases, hematologic toxicities were managed by dose reduction. Infections were reported in 11 patients (35%) overall and at grade 3/4 in six patients (19%), the most common being infections of the urinary tract (any grade in 3/31 patients (10%)).

Increases in aspartate aminotransferase (AST) and alanine aminotransferase (ALT) levels were frequent (Table 3). One patient in the 500 mg group had grade 3 ALT and AST elevations concurrent with a gastric ulcer perforation; the AST and ALT elevations improved to grade 1 following treatment of the gastric ulcer. Another patient in the 300 mg group with pre-existing intermittent mild transaminase elevations and polyarthritis developed submassive hepatic necrosis with hepatic failure during cycle 3 while receiving a reduced fedratinib dose of 200 mg. Grade 4 ALT and AST elevations, and hyperbilirubinemia were recorded. Recovery of liver function and return of liver function tests to normal ranges were noted after treatment discontinuation. An independent panel of liver experts reviewed this case and considered Hy's law criteria were met. Lipase and amylase elevations were also frequent. Grade 3 lipase elevations were observed in two patients in each dose group and were reversible to grade 1 or below. No instances of clinical pancreatitis or grade 3/4 amylase elevations were recorded. Increases in serum creatinine were also frequent, with all such events of grade 1/2 in severity.

Serious TEAEs were reported in six, three and five patients in the 300, 400 and 500 mg dose groups, respectively. The most common serious TEAEs were anemia (*n=*3) and nausea (*n=*2). One case of Wernicke's encephalopathy (WE) was reported in a 70-year-old female patient who had received study drug at 500 mg/day. The event was originally reported 60 weeks after initiation of fedratinib treatment as ischemic stroke confirmed by MRI. A persistent cognitive deficit was recognized in this patient over the following weeks and a subsequent MRI was consistent with WE. An independent expert safety panel, set up to investigate similar events in other fedratinib clinical trials, later confirmed the diagnosis as WE. Potential risk factors for WE in this patient included a history of chronic diarrhea, significant weight loss and cachexia predating study treatment. Intravenous thiamine supplementation led to some improvement in the neurologic symptoms but confusion persisted at last follow-up. PK analysis of samples collected earlier in the study indicated that fedratinib plasma levels in this patient were higher than the study population average.

Two patients died during the study, one in the 300 mg group 85 days after treatment discontinuation (reasons unknown) and one in the 500 mg group 36 days after treatment discontinuation (disease progression).

## Discussion

In this study, once-daily dosing with the JAK2-selective inhibitor fedratinib led to clinically significant and durable improvements in splenomegaly and symptom burden in patients with intermediate-2 or high-risk MF. Although the largest reduction in spleen volume was observed in the 500 mg group at 12 weeks, by 24 weeks, spleen volume reduction appeared greater with fedratinib in both the 400 and 500 mg groups compared with the 300 mg group. Similarly, the spleen response rates were higher in the 400 and 500 mg groups at 12 and 24 weeks, although the small numbers of patients in sample groups in this study precluded statistical analysis of differences in spleen response between dose levels. Spleen responses were durable and were maintained for a median of 251 days among the 21 patients remaining on treatment at the analysis cutoff date.

Changes in symptom burden were measured using the MPN-SAF, a validated tool for assessment of symptoms associated with MF.^23^ Fedratinib treatment led to improvement in the TSS, with reductions in the mean scores of each of the six key symptoms observed after 4 weeks of treatment. The improvement in symptoms was maintained, with a broadly similar reduction in individual symptom scores observed at 12 and 24 weeks. The degree of improvement in symptoms was broadly similar across the three doses.

Consistent with its mechanism of action in inhibiting JAK2, fedratinib treatment was associated with suppression of pSTAT3, with an approximate 50% reduction at 2 h after the initial dose, coincident with the observed t_max_ of fedratinib. This level of suppression was maintained at fedratinib trough level at steady state, indicating sustained JAK2 inhibition following repeated dosing.

In this study, no consistent changes were observed in *JAK2*V617F allele burden. This contrasts with the phase 1 trial, in which significant reductions in allele burden were observed following fedratinib treatment over 24 weeks, in particular among 23 patients with >20% allele burden at baseline.^20^ This discrepancy may be attributed to different assay methodologies used in the two studies,^20, 26, 27^ absence of higher-dose cohorts, and/or patient heterogeneity.

Many cytokines are abnormally expressed in patients with MF and this likely contributes to various constitutional symptoms associated with the disease.^14^ In this study, fedratinib significantly modulated 28 cytokines including TNF-α, whose downregulation correlated significantly with degree of spleen volume reduction. Similar findings have been reported with ruxolitinib treatment in patients with MF.^28^ TNF-α can facilitate expansion of the *JAK2*V617F clone,^29^ an activating mutation that can ultimately drive splenomegaly. Among other cytokines whose modulation correlated with spleen volume reduction, the anti-inflammatory and antifibrotic activity of adiponectin may be clinically relevant to MF. Adiponectin inhibits the expression of neural factor-κB target genes, leading to downregulation of both inflammatory cytokines (for example, TNF-α) and pro-fibrotic transforming growth factor-β signaling.^30, 31^ The increased production of adiponectin and decreased expression of inflammatory-related cytokines, as observed in this study, appear to dampen the inflammatory response and reduce symptom burden.

The safety profile of fedratinib observed in this study was generally consistent with that previously reported in the phase 1 study, in which the most common AEs were gastrointestinal events, fatigue, peripheral edema, dyspnea and treatment-related anemia.^20^ In contrast to the phase 1 study where 800 mg dosing was permitted,^20^ no grade 3/4 amylase elevations were observed at the 400 mg or 500 mg doses administered in this phase 2 study; asymptomatic grade 3/4 lipase elevations were recorded in a few patients and were reversible with dose reduction. Grade 1/2 liver function abnormalities were reported in some patients. Grade 4 ALT and AST elevations, and hyperbilirubinemia were recorded in one patient. However, recovery of liver function and return of liver function tests to normal ranges were noted after treatment discontinuation.

One patient in the 500 mg dosing cohort had an ischemic stroke with encephalopathy later suggested by MRI to be consistent with WE. Further identification of encephalopathy in other fedratinib-related studies prompted notification of investigators, institutional review boards/health committees and health authorities. Notably, seven cases of neurologic AEs were thought to be consistent with WE upon independent review, from the approximately 600 patients with MF, PV or solid tumors who received repeated doses of fedratinib in the fedratinib clinical program. Following a thorough risk–benefit analysis, once the cases of WE were recognized, the sponsor decided to discontinue clinical development of fedratinib.

In conclusion, these multicenter phase 2 clinical trial data confirm the robust clinical activity of fedratinib in patients with high-risk or intermediate-2 risk MF, in keeping with the phase 1 trial. However, the clinical benefit appeared to be counterbalanced by the occurrence of treatment-emergent encephalopathy, resembling WE, the mechanism and reversibility of which have not been resolved.

## Figures and Tables

**Table 1:** Baseline characteristics

*Characteristic*	*Fedratinib*		
	*300 mg*	*400 mg*	*500 mg*
	(n=*10)*	(n=*10)*	(n=*11)*
Median age, years (range)	57 (36–71)	66 (47–81)	67 (44–83)
			
*Sex,* n *(%)*			
* *Male	4 (40)	6 (60)	6 (55)
* *Female	6 (60)	4 (40)	5 (45)
			
*Disease subtype,* n *(%)*			
* *PMF	6 (60)	4 (40)	8 (73)
* *PPV-MF	3 (30)	4 (40)	1 (9)
* *PET-MF	1 (10)	2 (20)	2 (18)
* *High-risk status, *n* (%)	7 (70)	5 (50)	6 (55)
* *Prior hydroxyurea, *n* (%)	4 (40)	8 (80)	8 (73)
			
*JAK2V617F mutation status,* n *(%)*			
* *Positive	7 (70)	9 (90)	10 (91)
* *Negative	1 (10)	1 (10)	1 (10)
* *Unknown	2 (20)	0	0
* *RBC transfusion dependent, *n* (%)^a^	0	0	2 (18)
* *Median spleen volume (MRI), ml (range)	2603	2468	1616
	(1567–7503)	(1665–8265)	(654–3770)
* *Median MPN-SAF score at baseline (range)^b^	22.5 (1–34)	21.5 (12–39)	18.0 (4–40)

Abbreviations: MPN-SAF, Myeloproliferative Neoplasm Symptom Assessment Form; MRI, magnetic resonance imaging; PET-MF, post-essential thrombocythemia myelofibrosis; PMF, primary myelofibrosis; PPV-MF, post-polycythemia vera myelofibrosis; RBC, red blood cell.

a Transfusion dependency is defined as receiving an average of ⩾2 units of RBC transfusion per month over 3 months.^32^

b Median MPN-SAF score based on sum of six key constitutional symptoms (night sweats, pruritus, abdominal discomfort, abdominal pain, bone pain and early satiety).

**Table 2:** Spleen responses (intent-to-treat population)

	*Fedratinib*		
	*300 mg (*n=*10)*^a^	*400 mg (*n=*10)*	*500 mg (*n=*11)*
*Spleen volume reduction from baseline (%), mean (s.d.)*			
12 Weeks^b^	−30.3 (12.6)	−33.1 (19.0)	−43.3 (19.0)
24 Weeks	−26.6 (14.2)	−37.2 (22.5)	−41.1 (22.0)
			
*Spleen response,*^c^ *n (%)*			
12 Weeks	3 (30)	5 (50)	7 (64)
24 Weeks	3 (30)	6 (60)	6 (55)
48 Weeks	3 (30)	8 (80)	5 (45)

a Two patients discontinued treatment before week 12.

b Primary end point.

c Patients with a ⩾35% reduction in spleen volume from baseline.

**Table 3:** Summary of most common treatment-emergent AEs^a^

*AE,* n *(%)*	*Fedratinib*							
	*300 mg (*n=*10)*		*400 mg (*n=*10)*		*500 mg (*n=*11)*		*Total (*n=*31)*	
	*All grades*	*Grade 3/4*	*All grades*	*Grade 3/4*	*All grades*	*Grade 3/4*	*All grades*	*Grade 3/4*
*Nonhematologic AEs*								
Fatigue	8 (80)	3 (30)	7 (70)	1 (10)	8 (73)	0	23 (74)	4 (13)
Diarrhea	7 (70)	1 (10)	9 (90)	2 (20)	9 (82)	1 (9)	25 (81)	4 (13)
Nausea	7 (70)	1 (10)	7 (70)	1 (10)	10 (91)	0	24 (77)	2 (6)
Vomiting	6 (60)	2 (20)	7 (70)	1 (10)	8 (80)	0	21 (68)	3 (10)
Constipation	2 (20)	0	4 (40)	0	3 (27)	0	9 (29)	0
Peripheral edema	4 (40)	0	4 (40)	1 (10)	3 (27)	0	11 (35)	1 (3)
Dyspnea	2 (20)	0	4 (40)	1 (10)	3 (27)	0	9 (29)	1 (3)
Pain in extremity	2 (20)	1 (10)	3 (30)	0	2 (18)	0	7 (23)	1 (3)
Infections	2 (20)	0	3 (30)	2 (20)	6 (55)	4 (36)	11 (35)	6 (19)
								
*Hematologic AEs*^b^								
Anemia	10 (100)	6 (60)	10 (100)	5 (50)	11 (100)	7 (64)	31 (100)	18 (58)
Thrombocytopenia	5 (50)	2 (20)	5 (50)	1 (10)	7 (64)	2 (18)	17 (55)	5 (16)
Leukopenia	3 (30)	1 (10)	1 (10)	0	5 (45)	0	9 (29)	1 (3)
								
*Laboratory parameters*								
ALT	3 (30)	1 (10)	5 (50)	0	10 (91)	1 (9)	18 (58)	2 (6)
AST	4 (40)	1 (10)	8 (80)	0	10 (91)	1 (9)	22 (67)	2 (6)
Bilirubin	3 (30)	1 (10)	4 (40)	0	3 (27)	0	10 (32)	1 (3)
Creatinine	5 (50)	0	6 (60)	0	8 (73)	0	19 (61)	0
Amylase	4 (40)	0	3 (30)	0	5 (45)	0	12 (39)	0
Lipase	4 (40)	2 (20)	7 (70)	2 (20)	6 (55)	2 (18)	17 (55)	6 (19)

Abbreviations: AE, adverse event; ALT, alanine aminotransferase; AST, aspartate aminotransferase.

a Reported in >20% of patients across all dose groups.

b Laboratory evaluations.

## Notes

AP has received clinical trial support from Sanofi, Bristol-Myers Squibb, PharmaMar and JW Pharma. CJ has received research funding from J&J and Roche, and has provided consulting investigator advice to Sanofi. FL, CX and HC are employees of Sanofi. CL and GG were employees of Sanofi while the trial was being conducted and during the analysis of the data. MT has received research funding from Novartis, Bristol-Myers Squibb, Ariad and Deciphera, and has participated in speaker bureaus for Bristol-Myers Squibb, Ariad and Deciphera. The remaining authors declare no conflict of interest. All authors contributed to the analysis and interpretation of the data. All authors provided critical review of the manuscript during development, and approved the final version for submission.
