# Supplementary material for: A phase 2 randomized dose-ranging study of the JAK2-selective inhibitor fedratinib (SAR302503) in patients with myelofibrosis
Source: Blood Cancer J. 2015 Aug 7;5(8):e335–. doi: 10.1038/bcj.2015.63 (PMC4558588; doi:10.1038/bcj.2015.63)
Supplement: Supplementary Information [file bcj201563x1.doc]

**Supplementary information**

**METHODS**

Rules to stop or interrupt fedratinib treatment

Treatment was stopped in the event of unacceptable toxicity; disease progression (≥ 25% increase in spleen volume [magnetic resonance imaging measurement] compared with baseline or leukemic transformation [≥ 20% increase in peripheral blood blast levels over ≥ 8 weeks]); requirement for therapy or intervention precluded by the protocol; or patient withdrawal of consent.

Treatment interruption was mandated in the event of grade 4 thrombocytopenia or neutropenia; liver function test abnormalities (grade ≥ 3 alanine aminotransferase [ALT], aspartate aminotransferase [AST] or total bilirubin elevation); grade ≥ 3 nausea, vomiting, diarrhea, constipation or fatigue that did not respond to therapeutic measures within 48 h; any grade ≥ 3 nonhematologic/nongastrointestinal toxicity; or grade ≥ 2 peripheral neuropathy. Dose was resumed at a 100 mg/day decrement, except in cases of liver function test abnormalities. Dose re-escalation was permitted given that the lower dose was tolerated and stable for at least two treatment cycles.

Pharmacokinetic analysis

Fedratinib concentrations were determined using validated liquid chromatography tandem mass spectrometry in plasma samples collected at: days 1 and 29 (pre-dose and 1, 2, 3, 4, 6, 8 and 24 h post-dose); day 15 (pre-dose); and day 57 (pre-dose). Pharmacokinetic data were analyzed using WinNonlin software (WinNonlin Professional Version 5.2.1, Pharsight). Pharmacokinetic parameters assessed included: AUC_0–24_ (area under the plasma concentration versus time curve, calculated using the trapezoidal method from time 0 to 24 hours post-dose); C_max_ (maximum observed plasma concentration); t_max_ (time to reach C_max_); and R_ac_ (accumulation ratio calculated with R_ac_ = AUC_0–24_ [day 29]/AUC_0–24_ [day 1]).

Pharmacodynamic analyses

pSTAT3 inhibition

Granulocyte-colony stimulating factor (5 ng/ml)-stimulated phosphorylation of STAT3 was analyzed in peripheral blood leukocytes at day 1 (pre-dose, and 2, 6 and 24 h post-dose), day 15 (pre-dose) and day 29 (pre-dose). Levels were measured using a Phospho-STAT3 Whole Cell Lysate Kit (Meso Scale Discovery, Rockville, MD, USA) and normalized to total STAT3 determined using a Total STAT3 Sandwich ELISA Kit (Cell Signaling Technology, Danvers, MA, USA). Samples were analyzed at a central laboratory.

Allele burden

*JAK2*V617 mutant allele burden was measured in all patients at baseline. Changes in *JAK2*V617F allele burden were determined in genomic DNA from peripheral blood granulocytes using a validated quantitative polymerase chain reaction (allele-specific) assay^1^ at weeks 4, 12, 24, 36 and 48. The lower limit of detection of the assay was 0.05% *JAK2*V617F, and the lower limit of quantification was 0.5% *JAK2*V617F. The *MPL* gene was sequenced as a part of amplicon-based Illumina sequencing using primers from the Ion AmpliSeq^TM^ Comprehensive Cancer Panel (Life Technologies, Carlsbad, CA, USA) in patients’ baseline samples.

Cytokine analysis

The levels of 97 plasma cytokines (including cytokines, growth factors, hormones and other soluble proteins) were measured using microsphere-based immuno-multiplex assays (Rules Based Medicine, Austin, TX, USA) at baseline, and at the end of weeks 4, 8 and 12. The statistical significance of change in cytokine levels from baseline was calculated using an analysis of variance test, adjusted for multiple comparisons by the Hochberg procedure. The relationship between changes in cytokine levels and spleen volume was determined by a global nonparametric correlation analysis. Hierarchical clustering of patients was based on the regulated cytokine profiles.

**REFERENCE**

1. Lui F, Talpaz M, Pardanani A, Jamieson C, Gabrail N, Tefferi A, *et al*. Determination of JAK2V617F allele burden reduction in a Phase 2 study of patients with myelofibrosis treated with SAR302503 using a sensitive and robust allele-specific qPCR assay. *AACR American Association of Cancer Research*, April 6–10, 2013; Washington, DC, USA. Abstract No. LB-294.
