# Supplementary material for: A phase 2 randomized dose-ranging study of the JAK2-selective inhibitor fedratinib (SAR302503) in patients with myelofibrosis
Source: Blood Cancer J. 2015 Aug 7;5(8):e335–. doi: 10.1038/bcj.2015.63 (PMC4558588; doi:10.1038/bcj.2015.63)
Supplement: Supplementary Table S1 [file bcj201563x2.doc]

| **Table S1.** Change in *JAK2*V617F allele burden from baseline | | | |
| --- | --- | --- | --- |
| *Change from baseline in JAK2V617 allele burden (%)* | *Fedratinib* | | |
|  | *300 mg (*n *= 10)* | *400 mg (*n *= 10)* | *500 mg (*n *= 11)* |
| Week 12 Number of patients  Median (range) Mean (SD) | 4  –4.33 (–10.1 to 0.4) –4.59 (5.17) | 9  –0.41 (–17.3 to 0.2) –3.64 (5.88) | 9  –1.07 (–99.7 to 23.4) –12.52 (34.75) |
| Week 24 Number of patients  Median (range) Mean (SD) | 5  –7.22 (–17.6 to 0.5) –7.53 (7.77) | 9  –0.10 (–27.1 to 19.7) –2.33 (12.24) | 9  –6.50 (–68.8 to 29.1) –9.99 (26.04) |
| Week 36 Number of patients  Median (range) Mean (SD) | 4  –1.82 (–29.3 to 0.5) –8.12 (14.23) | 7  0.00 (–4.5 to 0.9) –1.00 (2.11) | 8  –6.22 (–68.8 to 49.7) –7.81 (32.33) |
| Week 48 Number of patients  Median (range) Mean (SD) | 4  –4.57 (–24.1 to 0.5) –8.19 (10.99) | 6  –0.05 (–21.9 to 2.0) –3.84 (8.99) | 8  –8.16 (–68.8 to 63.0) –7.79 (36.60) |
| Abbreviation: SD, standard deviation. | | | |
