# Supplementary material for: A phase 2 randomized dose-ranging study of the JAK2-selective inhibitor fedratinib (SAR302503) in patients with myelofibrosis
Source: Blood Cancer J. 2015 Aug 7;5(8):e335–. doi: 10.1038/bcj.2015.63 (PMC4558588; doi:10.1038/bcj.2015.63)
Supplement: Supplementary Table S2 [file bcj201563x3.doc]

| **Table S2.** Cytokine regulation at weeks 4, 8 and 12 | | | |
| --- | --- | --- | --- |
| *Cytokine* | *Fold change* vs *BL* | | |
|  | *Week 4* | *Week 8* | *Week 12* |
| **C-reactive protein** | –5.33 | –3.43 | –3.64 |
| **T-cell specific protein RANTES** | –2.23 | –2.98 | –3.54 |
| **EN-RAGE** | –2.10 | –2.70 | –3.49 |
| **Brain-derived neurotrophic growth factor** | –2.36 | –2.45 | –3.17 |
| **Thrombospondin-1** | –2.26 | –2.72 | –2.94 |
| **Matrix metalloproteinase-9** | –2.43 | –1.99 | –2.46 |
| **Calcitonin** | –2.20 | –1.76 | –2.11 |
| **Interleukin-1 receptor antagonist** | –2.07 | –2.21 | –2.10 |
| **Epithelial-derived neutrophil activating protein 78** | –1.54 | –1.81 | –2.08 |
| **Prostatic acid phosphatase** | –1.79 | –1.94 | –1.96 |
| **Tumor necrosis factor-α** | –1.76 | –1.71 | –1.91 |
| **Myeloperoxidase** | –2.32 | –1.55 | –1.75 |
| **Plasminogen activator inhibitor 1** | –1.63 | –1.72 | –1.69 |
| **MHC class 1 chain-related protein A** | –1.56 | –1.51 | –1.68 |
| E-selectin | –1.43 | –1.60 | –1.59 |
| CD40 ligand | –1.26 | –1.32 | –1.57 |
| **Interleukin-18** | –1.63 | –1.59 | –1.55 |
| Growth-regulated α protein | –1.17 | –1.37 | –1.55 |
| Interferon gamma-induced protein 10 | –1.52 | –1.39 | –1.35 |
| Pregnancy-associated plasma protein A | 1.53 | 1.21 | 1.17 |
| Carcinoembryonic antigen | 1.42 | 1.49 | 1.51 |
| Insulin | 1.18 | 1.35 | 1.52 |
| Myoglobin | 1.27 | 1.37 | 1.57 |
| Creatinine kinase-MB | 1.50 | 1.49 | 1.65 |
| **Adiponectin** | 1.71 | 1.74 | 1.71 |
| **Leptin** | 1.61 | 1.74 | 1.73 |
| **Ferritin** | 1.64 | 1.83 | 1.78 |
| **Erythropoietin** | 2.70 | 2.84 | 3.07 |
| Abbreviations: BL, baseline; EN-RAGE, extracellular newly identified receptor for advanced glycation end products binding protein; MHC, major histocompatibility complex; RANTES, regulated upon activation normal T cell expressed.  Cytokines in bold typeface had ≥ 1.5-fold regulation at all three time-points. *n =* 29 *P*-value is calculated using an analysis of variance test, adjusted for multiple comparisons by the Hochberg procedure. | | | |
