# Supplementary material for: A phase 2 randomized dose-ranging study of the JAK2-selective inhibitor fedratinib (SAR302503) in patients with myelofibrosis
Source: Blood Cancer J. 2015 Aug 7;5(8):e335–. doi: 10.1038/bcj.2015.63 (PMC4558588; doi:10.1038/bcj.2015.63)
Supplement: Supplementary Table S3 [file bcj201563x4.doc]

| **Table S3**. Cytokines with change in level significantly correlated with spleen volume reduction | | | | |
| --- | --- | --- | --- | --- |
| *Cytokine* | *Fold change at week 12 from baseline* | *Correlation between cytokine change at week 12* vs *spleen volume reduction* | | |
|  |  | *Spearman rho correlation coefficient* | *Raw* P*-value* | *FDR-adjusted* P*-value* |
| Prostatic acid phosphatase | –1.96 | 0.618 | 0.0006 | 0.0070 |
| Tumor necrosis factor-α | –1.91 | 0.543 | 0.0034 | 0.0292 |
| Carcinoembryonic antigen | 1.51 | –0.539 | 0.0037 | 0.0292 |
| Interleukin-18 | –1.55 | 0.513 | 0.0062 | 0.0389 |
| Myeloperoxidase | –1.75 | 0.507 | 0.0070 | 0.0389 |
| Adiponectin | 1.71 | –0.499 | 0.0080 | 0.0415 |
| Matrix metalloproteinase-9 | –2.46 | 0.483 | 0.0105 | 0.0472 |
| Creatinine kinase-MB | 1.65 | –0.482 | 0.0109 | 0.0472 |
| Myoglobin | 1.57 | –0.468 | 0.0138 | 0.0565 |
| EN-RAGE | –3.49 | 0.449 | 0.0187 | 0.0695 |
| Leptin | 1.73 | –0.403 | 0.0370 | 0.1255 |
| Ferritin | 1.78 | –0.392 | 0.0432 | 0.1404 |
| E-selectin | –1.59 | 0.388 | 0.0456 | 0.1421 |
| Abbreviation: EN-RAGE, extracellular newly identified receptor for advanced glycation end products binding protein. | | | | |
