# Supplementary figures and images for: A phase 2 randomized dose-ranging study of the JAK2-selective inhibitor fedratinib (SAR302503) in patients with myelofibrosis
Source: Blood Cancer J. 2015 Aug 7;5(8):e335–. doi: 10.1038/bcj.2015.63 (PMC4558588; doi:10.1038/bcj.2015.63)

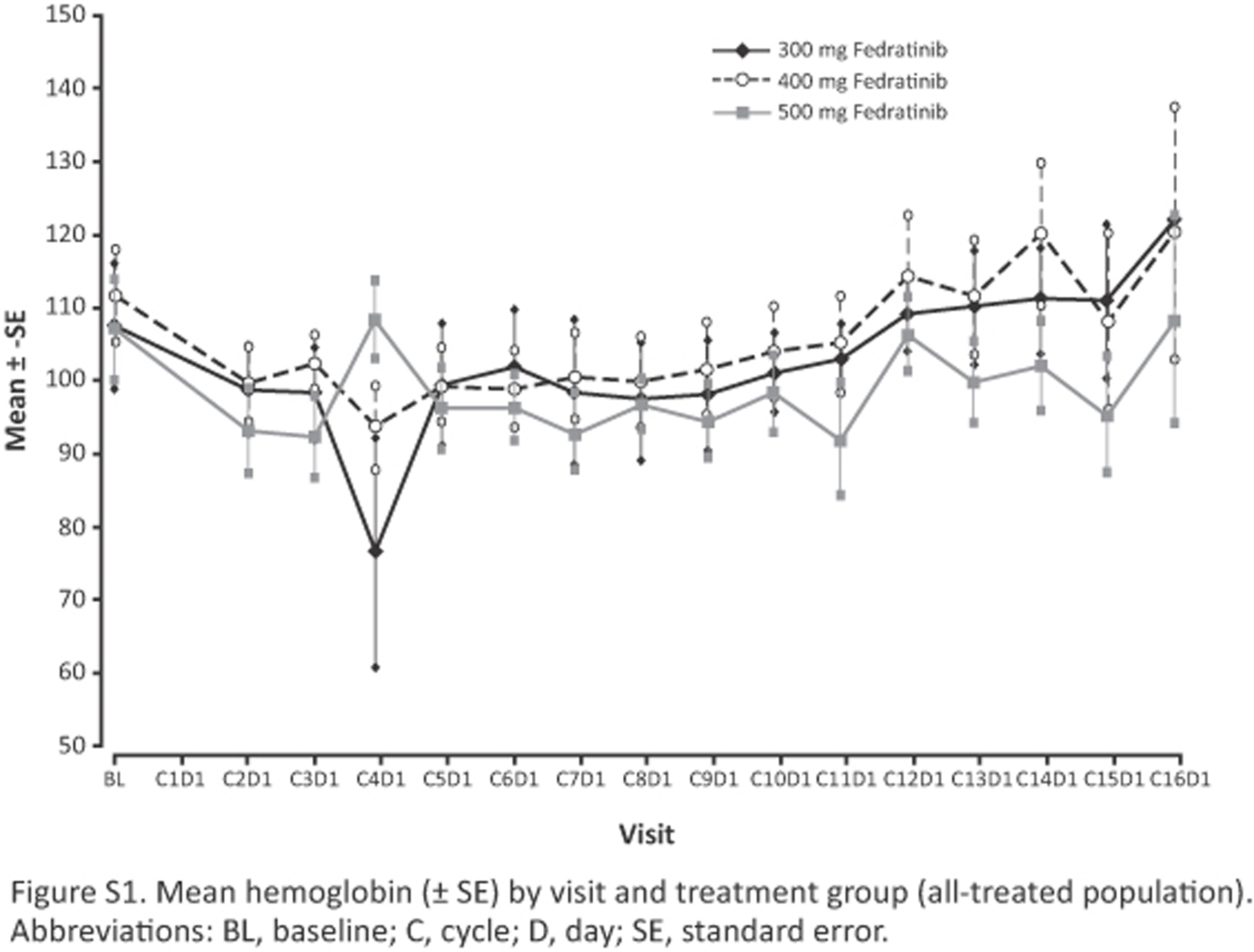

Supplement: Supplementary Figure S1 [file bcj201563x5.tif]

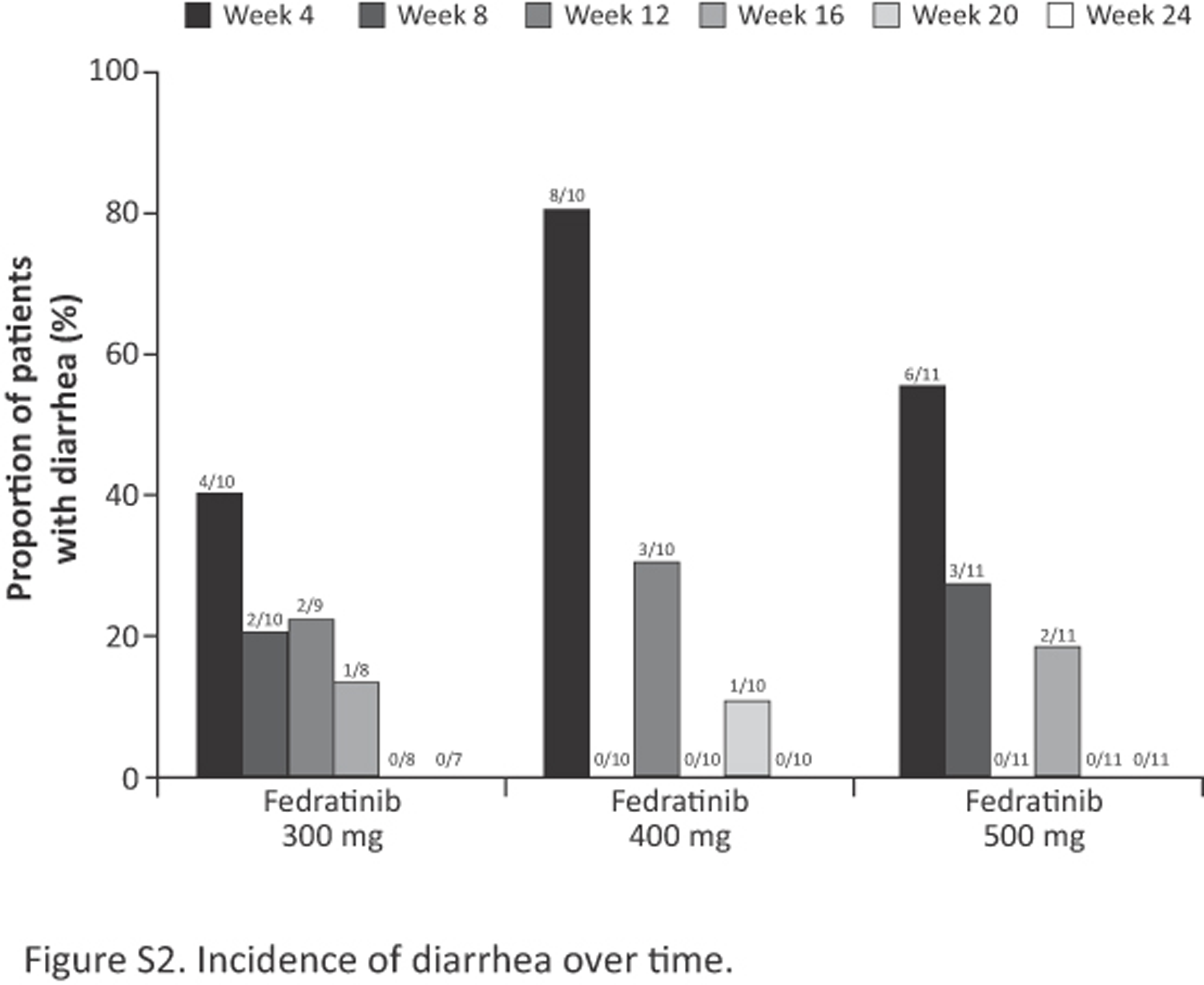

Supplement: Supplementary Figure S2 [file bcj201563x6.tif]
